# Solitary palmar adult xanthogranuloma^☆^

**DOI:** 10.1016/j.abd.2022.10.014

**Published:** 2023-11-22

**Authors:** Laura Serra-García, Cristina Carrera, Priscila Giavedoni, Constanza Riquelme-Mc Loughlin

**Affiliations:** Department of Dermatology, Hospital Clínic de Barcelona, Universitat de Barcelona, Barcelona, Spain

*Dear Editor,*

A 45-year-old woman with a history of breast cancer two years earlier, consulted for an enlarging asymptomatic lesion on the right palm which had appeared 4 months prior to consultation. During physical examination, she presented a 5 mm dome-shaped yellowish papule with a desquamative peripheral rim (Fig. 1A). Dermoscopy revealed homogeneous symmetric yellow structure-less areas with a central crust and pink surrounding rim (‘setting sun’ pattern) (Fig. 1B). Skin ultrasound revealed a round hypoechoic lesion occupying dermis and hypodermis, with multiple peripheral small vessels (Fig. 2). A benign adnexal tumor was suspected, but a cutaneous metastasis needed to be ruled out. Complete lesion excision revealed a dermal proliferation of mononucleated foamy histiocytes and Touton’s giant cells, which were positive for CD68 and negative for S100 and CD1a immunohistochemical stains (Fig. 3). A diagnosis of solitary adult xanthogranuloma was made. After complete excision, the lesion has not recurred. The patient remains in complete remission of her breast carcinoma.

**Figure 1:**
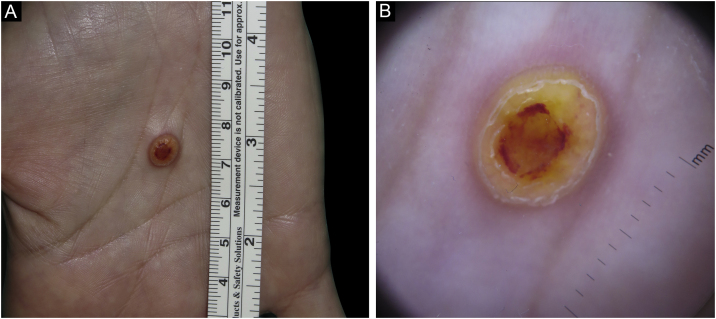
Solitary adult xanthogranuloma of the palm. (A) Yellowish 5 mm dome-shaped papule with central erythematous crust and a desquamative erythematous peripheral rim. (B) Dermoscopy showing homogeneous symmetric yellow structure-less areas with a central crust and pink surrounding rim (‘setting sun’ pattern).

**Figure 2:**
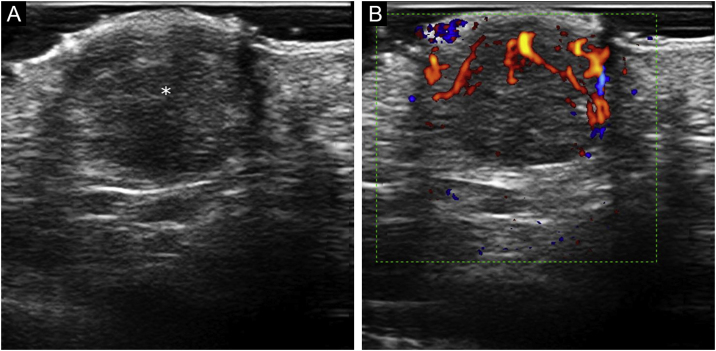
Sonographic characteristics of volar adult xanthogranuloma. (A) Grayscale sonogram (transverse view, right palm) showing a well-defined hypoechoic, dermal, and hypodermal nodule (asterisk) displacing the epidermal layer upwards; (B) Color Doppler sonogram of the same lesion showing inner vascularity.

**Figure 3:**
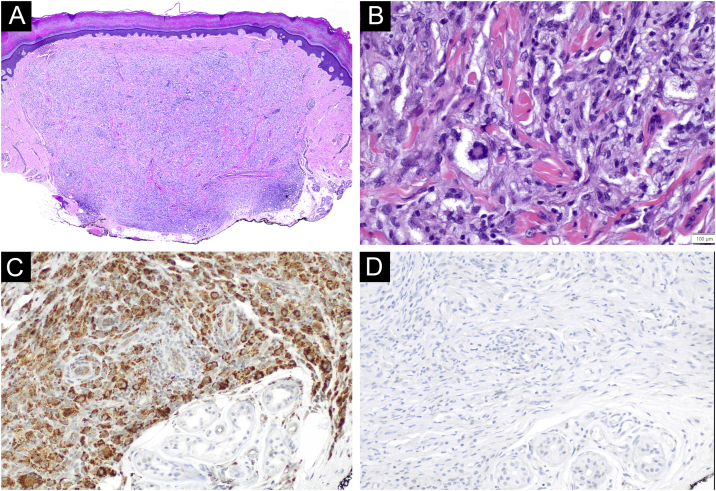
Histological characteristics of volar adult xanthogranuloma. (A and B) Dermal proliferation of mononucleated foamy histiocytes and Touton’s giant cells (Hematoxylin & eosin, ×40 and ×400, respectively). (C and D) Immunohistochemical stains revealing CD68 positive and CD1a negative foamy histiocytes, respectively (magnification ×200).

Adult xanthogranuloma is a subtype of non-Langerhans Cell Histiocytosis (LCH) belonging to the xanthogranuloma family, classified into the C (“Cutaneous”) group of the revised classification of histiocytoses.^1^ Adult xanthogranuloma is histologically identical to Juvenile Xanthogranuloma (JXG), the most common non-LCH. It usually presents in males in the first two decades of life, and the most common presentation is a solitary asymptomatic papule or nodule varying from 5 mm to 1‒2 cm in size, with a translucent, smooth, yellowish to brownish surface and occasional telangiectasias. In adults, this lesion tends to persist over time, rather than involute with time as in children. Adult and juvenile xanthogranulomas are usually located in the head and neck region, followed by the trunk and extremities; however, all cutaneous locations have been described, including palms and soles.^2^ Case reports including cases of JXG on the volar surfaces (palms and soles) have described unusual colors appearing in this location, such as dull red or flesh-colored, and a well-defined hyperkeratotic peripheral rim upon dermoscopy.^3^ Typical dermoscopic findings of adult and juvenile xanthogranuloma include a yellow/orange and red/pink homogeneous background, often referred to as the ‘setting sun’ pattern.^4^ Clinical differential diagnosis includes several benign and malignant tumors, such as Spitz nevus, dermatofibroma, adnexal neoplasms, molluscum contagiosum, hemangioma, basal cell carcinoma, amelanotic melanoma, and lymphomas. On volar locations, the differential diagnosis must include volar neoplasms such as eccrine poroma, pyogenic granuloma, digital fibrokeratoma, nevi, viral verruca, amelanotic melanoma, and solitary reticulohistiocytoma.^3^ There are few reports describing the sonographic characteristics of xanthogranulomas. These present as a well-defined hypoechoic dermal nodule, with thin low-velocity arterial vascularity (maximum peak systolic velocity, 6.5 cm/s) detected internally on color Doppler ultrasonography. No posterior enhancement or acoustic shadowing artifact has been reported.^5^

In this case, a cutaneous metastasis had to be ruled out given the patient’s past medical history. Excluding melanoma, breast carcinoma is the most frequent source of cutaneous metastasis in women.^6^ Because of its high incidence, breast carcinoma cutaneous metastases are the cutaneous metastases most frequently evaluated by dermatologists. The most common presentation of breast cancer cutaneous metastasis is an asymptomatic, skin-colored, or pink-brown nodule located on the chest wall or abdomen which might also occur on the extremities. A wide spectrum of clinical presentations has been described in cutaneous breast cancer metastases, which might mimic a wide variety of benign skin lesions, such as acral fibrokeratoma, pyogenic granuloma, follicular cysts, dermatofibromas or hemangioma, included in the differential diagnosis of adult xanthogranuloma. Ultrasound examination has proven to be a useful tool to support the diagnosis of cutaneous metastasis of melanoma.^7^ There is less evidence supporting the use of ultrasonography for the detection of cutaneous metastasis of solid tumors, however, this technique has been shown to aid in the diagnosis of malignant nodular and subcutaneous lesions in small series.^8^, ^9^ Sonographic characteristics suggestive of cutaneous metastases include polycyclic shape and hypervascularity with multiple peripheral poles and internal vessels.^10^ Cutaneous metastases herald a poor prognosis, hence the importance of dermatology in determining an early diagnosis.

## Financial support

None declared.

## Authors’ contributions

Laura Serra-García: Effective participation in critical review of the literature, drafting and editing of the manuscript and approval of the final version of the manuscript.

Cristina Carrera: Intellectual participation in the propaedeutic and/or therapeutic conduct of the studied cases; critical review of the manuscript; approval of the final version of the manuscript.

Priscila Giavedoni: Intellectual participation in the propaedeutic and/or therapeutic conduct of the studied cases; critical review of the manuscript; approval of the final version of the manuscript.

Constanza Riquelme-Mc Loughlin: Effective participation in research orientation; intellectual participation in the propaedeutic and/or therapeutic conduct of the studied cases; critical review of the manuscript; approval of the final version of the manuscript.

## Conflicts of interest

None declared.
